# Carnot Cycle and Heat Engine: Fundamentals and Applications

**DOI:** 10.3390/e22030348

**Published:** 2020-03-18

**Authors:** Michel Feidt

**Affiliations:** Laboratory of Energetics, Theoretical and Applied Mechanics (LEMTA), URA CNRS 7563, University of Lorraine, 54518 Vandoeuvre-lès-Nancy, France; michel.feidt@univ-lorraine.fr

After two years of exchange, this specific issue dedicated to the Carnot cycle and thermomechanical engines has been completed with ten papers including this editorial.

Our thanks are extended to all the authors for the interesting points of view they have proposed: this issue confirms the strong interactions between fundamentals and applications in thermodynamics.

Regarding the list of published papers annexed at the end of this editorial, it appears that six papers [1,2,3,4,5,6] are basically concerned with thermodynamics concepts. The three others [7,8,9] employ thermodynamics for specific applications. Only the papers included in the special issue are cited and analyzed in this editorial.

In “OTEC Maximum Net Power Output Using Carnot Cycle and Application to Simplify Heat Exchanger Selection”, Fontaine et al. [7] consider the maximum net power for the OTEC system using the Carnot cycle to simplify heat exchanger selection. The paper “Energy and Exergy Evaluation of a Two-Stage Axial Vapor Compressor on the LNG Carrier”, by Poljak et al. [8], particularizes energy and exergy evaluation to a component of a system: a two-stage axial vapor compressor for LNG application. In “Second Law Analysis for the Experimental Performances of a Cold Heat Exchanger of a Stirling Refrigeration Machine”, Djetel-Gothe et al. [9] also use second law analysis to quantify experimental performances of a cold heat exchanger of a Stirling refrigerator system.

The first conclusion is that we must enlarge the subject from engines to reverse cycle machines, and particularly those dedicated to low temperature applications. This is confirmed by the paper “Global Efficiency of Heat Engines and Heat Pumps with Non-Linear Boundary Conditions” [3] which is concerned both with efficiency of heat pumps and heat engines. This paper establishes the connection between applications and concepts, mainly global efficiency in this case. This concept is fruitful due to its non-dimensional form. Lundqvist and Öhman [3] use a black box method to compare thermal efficiencies of different scale and type of engines and heat pumps. The influence of boundary conditions is exemplified. Using FTT (Finite Time Thermodynamics) and Max power cycle approaches easily enable a black box modeling with various (linear or not) boundary conditions.

Two papers give respectively a short history regarding efficiency at maximum power and an analysis of the methodology used in modeling and optimization of Carnot engines [2,6]. It appears that in any of the cases specific physical dimensions are correlated to the choice of objective function and constraints. This is why we preconize the acronym FDOT (Finite physical Dimensions Optimal Thermodynamics) instead of FTT. In “Progress in Carnot and Chambadal Modeling of Thermomechanical Engines by Considering Entropy Production and Heat Transfer Entropy”, Feidt and Costea [6] details some progress in Carnot and Chambadal modeling of thermomechanical engines by considering entropy production as well as heat transfer entropy.

The paper by Gonzalez-Ayala et al. [1] has the same philosophy as the paper by Feidt and Costea [6] but using finite time heat engine models compared to low dissipation models. The proposed models take account of heat leak and internal irreversibilities related to time; the maximum power (MP) regime is covered. Upper and lower bounds of MP efficiency are reported depending on the heat transfer law.

The paper by Xue and Guo [4] is more exotic. It re-examines the Clausius statement of the second law of thermodynamics. This paper introduces an average temperature method.

We hope to have the opportunity to continue to report on the progress of the subject in the near future, extending the subject to reverse cycle configurations.
